# The relationship between postmenopausal women’s body image and the severity of menopausal symptoms

**DOI:** 10.1186/s12889-021-11643-6

**Published:** 2021-08-30

**Authors:** Soheila Nazarpour, Masoumeh Simbar, Hamid Alavi Majd, Zahra Jafari Torkamani, Khadijeh Dodel Andarvar, Fatemeh Rahnemaei

**Affiliations:** 1 Department of Midwifery, Chalous Branch, Islamic Azad University, Chalous, Iran; 2 Midwifery and Reproductive Health Research Center, Shahid Beheshti University of Medical Sciences, Tehran, Iran; 3 Department of Midwifery and Reproductive Health, School of Nursing and Midwifery, Shahid Beheshti University of Medical Sciences, Vali-Asr Avenue, Cross of Vali-Asr and Neiaiesh Highway, Opposite to Rajaee Heart Hospital, Tehran, 1996835119 Iran; 4 Department of Biostatistics, School of Paramedicine, Shahid Beheshti University of Medical Sciences, Tehran, Iran

**Keywords:** Menopause, Body image, Menopausal symptoms, Socio-demographic factors

## Abstract

**Background:**

Body image is the main element of an individual’s personality that may be influenced by many factors during menopause. We aimed to assess the relationship between postmenopausal women’s body image with the severity of menopausal symptoms.

**Methods:**

This was a cross-sectional study on 300 postmenopausal women, aged 45 to 65 years old, in Tehran, Iran. We recruited the samples using the multi-stage sampling method. Tools for data collection were: 1) the Menopausal Rating Scale (MRS), 2) the Fisher’s Body Image questionnaire and 3) a socio-demographic questionnaire. We analyzed data using the independent samples *t*-test, Pearson correlation coefficient, Spearman’s correlation coefficient, and multiple linear regression tests.

**Results:**

Three hundred women aged 55.11 ± 3.99 years old, participated in the study. Mean scores for body image and MRS were 163.07 ± 21.17 (Range: 46–230) and 16.45 ± 8.38 (Range: 0–44), respectively. About 50% of women had severe symptoms of menopause (MRS score ≥ 17). There was a negative correlation between the total score and the score of all dimensions of body image with the total score and all dimensions of MRS (*P* < 0.001). There were also significant relationships between women’s body image with: their education (*P* < 0.001, r = 0.20) the spouse’s education (*P* < 0.001, r = 0.26), adequacy of monthly household income (*P* < 0.001, r = 0.32), marital status (*P* = 0.36), their occupation (*P* = 0.007) and housing status (*P* = 0.012). There was also a significant negative correlation between women’s lower body organs image with the number of children (*P* = 0.017, r = − 0.14). According to the multiple linear regressions model, severity of menopausal symptoms (Beta = − 0.45, *P* < 0.001) and adequacy of monthly household income (Beta = 0.15, *P* = 0.005) are the significant related factors with postmenopausal women’s body image.

**Conclusions:**

Body image is correlated with menopausal symptoms of women during menopause. Therefore, it seems that interventions aimed at relieving the annoying symptoms of menopause can help to improve their body image. Also, body image could be influenced by some socio-demographic factors which should be considered in menopause health promotion programs.

**Supplementary Information:**

The online version contains supplementary material available at 10.1186/s12889-021-11643-6.

## Background

Body image is a multidimensional, subjective and dynamic concept that encompasses a person’s perceptions, thoughts, and feelings about their own body [1]. It is one of the main elements of an individuals’ personality [2, 3]. Cash and Pruzinsky defined body image as perceptions, thoughts, and feelings associated with the body and bodily experience [4]. Positive body image is also different from negative body image and it is multifaceted (including body appreciation, body acceptance/love), holistic, stable and malleable, protective, linked to self-perceived body acceptance by others; and shaped by social identities [5]. Body image is a person’s perception of their physical self and the thoughts and feelings, positive, negative, or both, which result from that perception [6].

Several factors are shown to influence body image. These factors are dependent on the cultural and economic condition of every community [7, 8]. Several studies showed a relationship between body image with age [8, 9], gender [9, 10], marital status [9], educational level [9, 11], anxiety and depression [7, 12], social phobia [13], smoking and/or alcohol use [14], interpersonal sensitivity [15], obsessed behaviors [15], nutritional deficiencies [7], body mass index [10, 16, 17], low level of physical exercises [18], sexual orientation [19] sexual dysfunction and dyspareunia among women [20].

A study on 75,256 postmenopausal women showed a prevalence of 83% of body image dissatisfaction [16]. Postmenopausal period is a period of life that may influence the body image of women. Body changes with the increasing age of women may influence their body image [21] and may lead to a negative body image [22]. Majority of menopausal women experience symptoms of menopausal syndrome such as flushing, atrophic vaginitis, sexual dysfunction, insomnia, fatigue, musculoskeletal problems, cardiovascular symptoms, breast atrophy, change in shape, wrinkles in the skin, and the signs of elderly that may affect their body image [23–25]. Abbas et al. (2020) showed an association between health problems and psychological problems such as stress and anxiety [26]. It seems menopausal physical symptoms may lead to psychological problems such as depression and anxiety, and these problems could influence on body image of menopausal women [12]. While inappropriate perception and negative body image may lead to psychosomatic problems in postmenopausal women [27], positive body image improves self-confidence and satisfactory sexual life of postmenopausal women [28].

Respecting the possible effects of some postmenopausal complications on women’s body image and so decreasing their quality of life, we aimed to assess the relationship between postmenopausal women’s body image with the severity of menopausal symptoms.

## Methods

### Study design

This was a cross-sectional study. Three hundred women with an average age of 55.11 ± 3.99 years, with minimum to maximum age 45 to 65 years participated in the study. They were selected from attendees to health care centers of Tehran, Iran. These centers provide health services on an outpatient basis and are covered by the country’s medical universities and the Ministry of health. The study was conducted from March to July 2020. The participants were non-surgical or non-premature menopause women. They have no history of physical or psychological disorders, no severe stress, no use of herbal or chemical estrogens. Menopause is defined as the time when there are no menstrual periods for 12 previous consecutive months, with no other biological or physiological cause [29]. According to this definition, we included 45–65 years old women without any menstrual period for at least more than 12 consecutive months.

Subjects of the study were recruited by using the multi-stage probability-cluster sampling method. In cluster sampling, researchers divide a population into smaller groups known as clusters. They then randomly select among these clusters to form a sample. Cluster sampling is a method of probability sampling that is used to study large populations, particularly those that are widely geographically dispersed. Therefore, we selected the four geographic regions of Tehran as the first stage of clustering. Then, the health centers were randomly selected as the second stage of clustering. At the last stage, quota sampling was performed to select the subjects of the study. Written informed consent was obtained from every participant.

### Measures

The tools for data collection were; 1) a Socio-demographic questionnaire, 2) the translated version of the Menopause Rating Scale (MRS) [30], and 3) Fisher’s body image questionnaire [31].

*The socio-demographic questionnaire* was developed for this study by the researchers and had 10 questions about personal and social characteristics of participants including age, age of menopause, the number of children, educational level of women and their husbands, marital status, occupation of women and their husbands, housing status and adequacy of monthly household income. Adequacy of monthly household income was scored from 1 to 3 for “not adequate”, “adequate” and, “adequate and saving” choices (Supplementary file 1).

*The Menopause Rating Scale (MRS)* is an international, standard scale for rating the severity of menopausal symptoms. It is designed in three sections of somatic (vasomotor), psychological, and urogenital symptoms. The questionnaire consists of 11 questions, the responses to which are scored to the severity of the symptoms with a 5-point Likert scale, including: “none” (0), “mild” (1), “moderate” (2), “severe” (3) and “very severe” (4). A total score equal to or more than 17, is indicating severe menopausal symptoms [32]. The content and face validity of the MRS questionnaire were confirmed in a previous study [33]. The reliability of the MRS was assessed by the test-retest method and calculating Cronbach’s α and ICC of 0.933 and 0.977, respectively [33].

*Fisher’s Body Image questionnaire* was developed by Fisher [31]. The questionnaire includes 46 items which scores 1 to five (completely unsatisfied = 1; mostly unsatisfied = 2; moderate = 3; mostly satisfied = 4; completely satisfied = 5) (Ranges 46 to 230). Higher scores show more satisfaction with body image. The questionnaire was validated in Iran [34, 35]. The stability of the questionnaire was demonstrated by test-retest and Pearson correlation coefficient of more than 0.81. Internal consistency of the questionnaire was also shown by Cronbach’s α 0.93 and half-splitting correlation coefficient of 0.91 [34]. Nazarpour and Khazai calculated the reliability of this questionnaire by using Cronbach’s α, Spearman’s coefficient, and Guttman half splitting correlation coefficients equal to 0.918, 0.861, and 0.861, respectively [36].

All the questionnaires were completed using face-to-face interviews.

### Statistical analyses

The data were analyzed using the SPSS version 23 (SPSS Inc., Chicago, IL, USA). The continuous variables were checked for normality using the one-sample Kolmogorov-Smirnoff test. The correlation was measured by the Pearson correlation test (for variables with normal distribution), and Spearman’s correlation test (for variables with abnormal distribution and ordinal variables), and independent samples *t*-test.

Also, multiple linear regression was used to assess associated factors with body image. The assumption for the multiple linear regression model was that body image MRS and some socio-demographic factors are associated with body image as the outcome and dependent variable. All socio-demographic variables were included in the first block and MRS in the second block.

The socio-demographic factors that entered the model were: age, number of children, duration of menopause, marital status, occupation of women, educational level of women and her husband, their housing status, the adequacy of monthly household income. Because, they have been shown by other studies to be related to body image [8, 11, 37].

*A P*-value less than 0.05 was considered statistically significant.

## Results

### Description of the study sample

Three hundred women with an average age of 55.11 ± 3.99 (Mean ± SD) years old, with minimum to maximum age 45 to 65, participated in the study. The duration of menopause was 5.80 ± 4.42 months. The mean number of children was 3.34 ± 1.63. Ninety-five percent of women were married and 80% were housewives, 16% of women were illiterate and 16.3% had academic education. Fifty-two percent of participants had employed husbands, and remaining women had unemployed- or retired- husbands, and 77.3% of the subjects had housing ownership status. Socio-demographic characteristics of women are shown in Table 1.

**Table 1:** Socio-demographic characteristics of postmenopausal women participated in the study (*N* = 300)

Variables	Mean ± SD/n (%)
Age (years)	55.11 ± 3.99
Duration of menopause^a^ (months)	5.80 ± 4.42
Number of children	3.24 ± 1.63
Occupation	
Housewife or retired	262 (87.3)
Employed	38 (12.7)
Education	
Illiterate	48 (16.0)
Diploma and under diploma	203 (67.7)
Higher diploma	49 (16.3)
Marital status	
Married	286 (95.3)
Single/ Widow/ Divorced	14 (4.7)
Occupation of Husband	
Employed	156 (52.0)
Retired or unemployed	144 (48.0)
Education of husband	
Illiterate	31 (10.3)
Diploma and under diploma	216 (72.0)
Higher diploma	53(17.7)
Housing status	
The owner	232 (77.3)
Non-owner	68 (22.7)
The adequacy of monthly household income	
Less than adequate	130 (43.3)
Adequately or with saving	170 (56.7)

^a^Menopause is defined as the time when there have been no menstrual periods for 12 consecutive months;

The mean score of body image was 163.07 ± 11.50 (63.63 ± 11.50%). The highest and lowest scores were shown for head and face (67.36%) and lower organs (58.43%), respectively. Mean scores of body image and its domains are presented in Table 2.

**Table 2:** Mean and standard deviation of scores for women’s body image and severity menopausal symptoms

	Score	
Body Image	Mean ± SD (46–230)^a^	Mean ± SD (0–100)^a^
Head & face (12–60)	44.33 ± 5.44	67.36 ± 11.33
Upper limbs (10–50)	34.91 ± 6.03	62.27 ± 15.08
Lower limbs (6–30)	20.02 ± 4.27	58.43 ± 17.80
Overall	64.02 ± 8.48	63.92 ± 11.78
Total Body Image	163.07 ± 21.17	63.63 ± 11.50
MRS	Mean ± SD (0–44)	Mean ± SD (0–100)
Somatic	6.46 ± 3.66	40.42 ± 22.87
Psychological	6.31 ± 4.04	39.42 ± 25.25
Urogenital	3.67 ± 2.74	30.61 ± 22.87
Total score	16.45 ± 8.38	37.38 ± 19.04

^a^Total possible range of scores

The mean score for MRS was 37.38 ± 19.04%. The most severe symptoms of MRS were shown in the somatic domain (40.42%) and the lowest score was shown for the urogenital domain (30.61%). Severe symptoms (total score ≥ 17) were reported in 49.0% (147) of women. Mean scores of MRS and its domains are presented in Table 2.

### Correlations

Finding showed that total score and the dimensions of body image have significant negative correlations with total score and all domains of MRS (Table 3).

**Table 3:** The correlations between Menopause Rating Scale (MRS) and the socio-demographic factors with women’s body image

Variables	Body image (four dimensions and total scores).				
	Head & face	Upper limbs	Lower limbs	Overall	Total score
	*r*	*r*	*r*	*r*	*r*
The severity of menopausal symptoms; Test: Pearson correlation coefficient.					
Somatic	− 0.23^***^	− 0.35^***^	− 0.37^***^	−0.41^***^	−0.40^***^
Psychological	− 0.33^***^	−0.46^***^	− 0.40^***^	− 0.47^***^	− 0.49^***^
Urogenital	−0.30^***^	− 0.21^***^	− 0.20^***^	− 0.27^***^	−0.29^***^
Total score	− 0.36^***^	−0.45^***^	− 0.42^***^	−0.49^***^	−0.51^***^
The socio-demographic factors; Test: Spearman’s correlation coefficient.					
Age	0.05	0.05	−0.05	0.11	0.09
Duration of menopause	0.04	0.02	0.01	0.04	0.05
Number of children	− 0.02	−0.03	− 0.14^*^	0.04	−0.03
Education	0.17^**^	0.21^***^	0.23^***^	0.13^*^	0.20^***^
Education of Husband	0.23^***^	0.27^***^	0.26^***^	0.19^**^	0.26^***^
The adequacy of monthly household income	0.27^***^	0.33^***^	0.29^***^	0.27^***^	0.32^***^

* *P* < 0.05. ** *P* < 0.01. *** *P* < 0.001

Results of the study also demonstrated a significant positive correlation between body image with the educational level of women (*P* < 0.001, r = 0.20) and with the husband educational level (*P* < 0.001, r = 0.26) and the adequacy of monthly household income (*P* < 0.001, r = 0.32). There was the same correlation between all dimensions of body image with the same above-mentioned characteristics. There was also a significant negative correlation between the numbers of children with a score of lower limbs body image (*P* = 0.017, r = − 0.14((Table 3).

*T*-test demonstrated higher scores for body image of married women comparing with widow/single women (*P* = 0.036), and also for women with housing ownership status compared to women with rental housing status (*P* = 0.012), and for employed women comparing to housewife/retired women (*P* = 0.007).

### Regression

Multiple linear regression analysis showed that the model can show 27.2% of the variance of total scores for body image. The model also showed that MRS is an associated factor with body image. Otherwise, with increasing one score of MRS, body image decreases by 1.15 units (*P* < 0.001). Also, the adequacy of monthly household income is an associated factor with body image. Otherwise, women with a monthly income had higher scores of body image (Table 4). The interactions between age, number of children, duration of menopausal period, marital status, and women’s occupation, educational level of women and her husband, housing status, adequacy of monthly household income were assessed. However, these interaction terms were not included in the final model as they were not statistically significant (Table 4).

**Table 4:** Multiple linear regression analysis for postmenopausal women’s body image

Variables	Beta	T	*P* value	95% Confidence Interval for B	
				Lower	Upper
MRS	−0.45	−8.47	< 0.001	−1.41	−0.88
Adequacy of monthly household income ^a^	0.15	2.83	0.005	0.85	4.75
**Adjusted R**^**2**^ **= 0.272 R**^**2**^ **= 0.277**					

^**a**^ Classification of the adequacy of monthly household income: 0 (Zero). Less than adequate, 1. Adequate or saving

## Discussion

The study showed the severity of the menopausal symptom and all of its dimensions have a negative correlation with body image. Also, regression analysis showed the severity of menopausal syndrome is an associated factor with women’s body image. By this finding, Włodarczyk & Dolińska-Zygmunt (2017) showed women with severe vasomotor and psychosomatic symptoms experience low self-confidence and body function [38]. Perimenopausal physical and psychological changes such as gaining weight, insomnia, flushing, and night sweats as well as elderly symptoms like changes in skin and hair, sexual dysfunction, and osteoporosis can change women’s attitudes and feelings about their body image [24, 39]. Ginsberg et al. (2016) found a negative correlation between high body mass index with positive body image which means obese postmenopausal women have usually dissatisfied body image [16].

There is evidence to suggest that women’s interpretation of their menopausal experiences relates to their attitude towards menopause and body image. For example, women with a negative attitude towards menopausal physical changes, like weight gain, think that makes them less sexually desirable [40]. A negative attitude towards menopause is associated with body dissatisfaction, self-objectification, appearance-related aging anxiety, and lower perceived attractiveness during the menopausal transition [40–42]. A study on 350 women in menopause showed that 74.4% of them feel losing attractiveness after menopause [43]. Therefore, menopausal symptoms are associated with a negative attitude about attractiveness, low self-esteem, and body image [24]. Negative perception about menopausal changes associated with low scores for self-efficacy [44]. Also, changes in the appearance of postmenopausal women are associated with body image concerns which in turn may influence their quality of life [45]. Also, appearance-related aging concerns and body comparison are two sociocultural factors that moderated the association between menopausal status and disordered body image concerns [46].

We showed a significantly strong negative correlation between urogenital symptoms as a dimension of menopausal symptoms with women’s body image and all of its dimensions. It is consistent with the results of other studies showing that women with urogenital symptoms such as urinary incontinence or dyspareunia are unsatisfied with their body image [20, 47]. In this accordance, another study also showed urogenital symptoms especially stress incontinence can have negative impacts on menopausal women’s body image [47] as well as on their psychological health [48, 49]. A comparative study showed women with positive body image had less vaginal moistures and so less orgasmic problem and dyspareunia and then more sexual satisfaction (Zamankhani, 2005).

The results also demonstrated a significant correlation between women’s body image with some socio-demographic factors such as the educational level of the women and their husband; their number of children, marital status, income, employment, and housing status in menopause. Although there were no similar studies to show these associations in menopausal women, some studies showing these correlations in other groups of people. For example, a study on 1200 men and women showed a negative correlation between the participants’ education with their body dissatisfaction [9]. Another study on 126 women with breast cancer also showed a positive correlation between educational levels with their body image [11]. Also, a study on 1841 women demonstrated a relationship between individual characteristics such as women’s education level with women’s attitude towards body image and management [37]. It seems high-level educated women manage their menopausal symptoms better than low-level educated women.

There was a positive correlation between monthly household incomes with the women’s body image. Also, the regression analysis showed that monthly household income is an associated factor with body image. Similar results were shown in other studies on women affected by breast cancer [11] or among other women [37]. It may be because monthly household incomes are more common among empowered educated, employed, and homeowner women who manage their menopausal symptoms and so have appropriate body image.

The results showed body image of employed women was also significantly higher than unemployed ones. A similar result was shown in women with breast cancer [11]. It was shown that employment impacts women’s attitude towards body management [37]. Also, Housing ownership has a positive impact on the body image of women in menopause. A similar finding was also demonstrated in another study among women in all age groups [37]. Therefore, the present study showed the positive relationships between body image with education, employment, income, and housing ownership in menopause. These factors are the indexes of women’s empowerment [50] and so the study shows the relationship between women’s empowerment with the body image of women during menopause. Therefore, it can be postulated that women’s empowerment could be related to women’s body image and self-esteem.

This study showed a negative correlation between the numbers of children with lower limbs body image. It seems that the high number of children associate with a high number of vaginal deliveries may lead to a negative lower body image. Besides, the finding of the present study showed that married women have lower scores of body image comparing to single/widow/separated women. It was reported that married women are more unsatisfied with their bodies comparing to single women [9]. So, marital status is an influencing factor on women’s attitude towards body management [37].

This was a cross-sectional study that is not able to assess the cause and effect relationship; however, we used regression analysis to find out factors associated with the body image of postmenopausal women as the dependent variable. The regression model demonstrated the two factors including the severity of menopausal symptoms and monthly household income, can be the factors associated with the women’s body image. Therefore, further analytical studies are necessary to assess the cause and effect relationship between the severity of menopausal symptoms with the women’s body image. This study was the first study showing the relationship between all dimensions of menopausal symptoms with all dimensions of body image in menopausal women. This study could be a base for analytical design studies and interventional studies to improve postmenopausal women’s health.

### Strengths and limitations

The strength of the study was that women were not using hormone replacement therapy or phytoestrogenic medicines and so the severity of menopausal symptoms was not affected by these treatments. Also, studies about the effective factors on the body image of menopausal women are rare and this study can be a base for future researches.

A limitation of the study was that the participants were from Tehran, and since sociocultural and economic factors may be effective on postmenopausal women’s body image, perhaps further studies are necessary to be conducted in other communities with different conditions. Another limitation of the present study could be that the cross-sectional study did not permit the assessment of cause and effect relationships between severity of menopausal symptoms and also other socio-demographic characteristics with body image. We also recruited the samples from the public primary health care services that rich women are rarely using these services. Some factors were not measured; for example, BMI was not measured as many postmenopausal women are not aware of the meaning of BMI, and also, we could not measure their weight and height in the health centers because of corona-raised limitations. However, the study might have gained by having actual measurements of body size and proportions correlative with subjective image perception.

These factors such as BMI, habits like smoking, and alcohol use dietary and physical activities are suggested to be considered in future studies. It should be also mentioned that in our previous studies that alcohol use and smoking are rare among this group of women. Also, they are sensitive questions in the country, that responses may not be reliable.

## Conclusion

Menopausal symptoms can influence on body image of women in menopause. Postmenopausal women’s education, employment, income, and housing ownership are positively correlated with body image. These factors are the indexes of women’s empowerment and so women empowerment can improve body image and self-esteem. Although it was a cross-sectional study that was not able to assess the cause and effect relationship; the regression analysis showed that severity of menopausal symptoms and monthly household income, can be associated with women’s body image. Therefore, further analytical studies are necessary to assess the cause and effect relationship between the severity of menopausal symptoms with the women’s body image. This study could be a base for further analytical design studies and future interventional studies to improve postmenopausal women’s health. Women empowerment through improving women’s education, employment, income, and housing condition should be considered for future policies and programs to promote women’s health during menopause.

## Supplementary Information

**Additional file 1.**

## Data Availability

The datasets used and/or analyzed during the current study are available from the corresponding author on reasonable request.

## Abbreviations

MRS: Menopausal Rating Scale
ICC: Intra-class correlation
SPSS 21: Statistical Package for the Social Sciences (version 21)
