# Supplementary material for: The relationship between postmenopausal women’s body image and the severity of menopausal symptoms
Source: BMC Public Health. 2021 Aug 30;21:1599. doi: 10.1186/s12889-021-11643-6 (PMC8404323; doi:10.1186/s12889-021-11643-6)
Supplement: Supplementary file 1 — Additional file 1. [file 12889_2021_11643_MOESM1_ESM.docx]

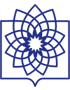


Shahid Beheshti University of Medical Sciences

Midwifery and Reproductive Health Research Center

Questionnaire code:

The research-assistant code:

Health center code

Date of completion of the questionnaire

**Socio-Demographic Information**

1- How old are you?

2- How many children (live) do you have?

3- How long have you been menopause? (Menopause refers to the time when at least 1 year has passed since the last menstrual period) Year

4- What is your current marital status?

🞎 Married

🞎 Single

🞎 Widowed

🞎 Divorced

5- What is your occupation?

🞎 Housewife

🞎 Government job

🞎Freelance job

🞎 Retired

🞎Self-employment

6- What is your level of education?

🞎 Illiterate

🞎 Primary and secondary school

🞎 High school and diploma

🞎 University education

7- What is your spouse's occupation?

🞎 Government job

🞎 Freelance job

🞎 Retired

🞎Unemployed

8- What is level of education of your husband?

🞎 Illiterate

🞎 Primary and secondary school

🞎 High school and diploma

🞎 University education

9- How is your housing situation?

🞎 Owner

🞎 Tenant

🞎 Living in a relative or father's home

10- Is your monthly income adequate for your needs?

🞎 I am completely in financial well-being

🞎 I can make a living by saving

🞎 I sometimes have financial problems

🞎 I always have financial problems

🞎 I am in complete poverty
